# Supplementary material for: Methemoglobinemia, Increased Deformability and Reduced Membrane Stability of Red Blood Cells in a Cat with a CYB5R3 Splice Defect
Source: Cells. 2023 Mar 24;12(7):991. doi: 10.3390/cells12070991 (PMC10093206; doi:10.3390/cells12070991)
Supplement: Supplementary file 1 [file cells-12-00991-s001.zip › cells-2249409-supplymentary-done.pdf]

Supplementary Materials

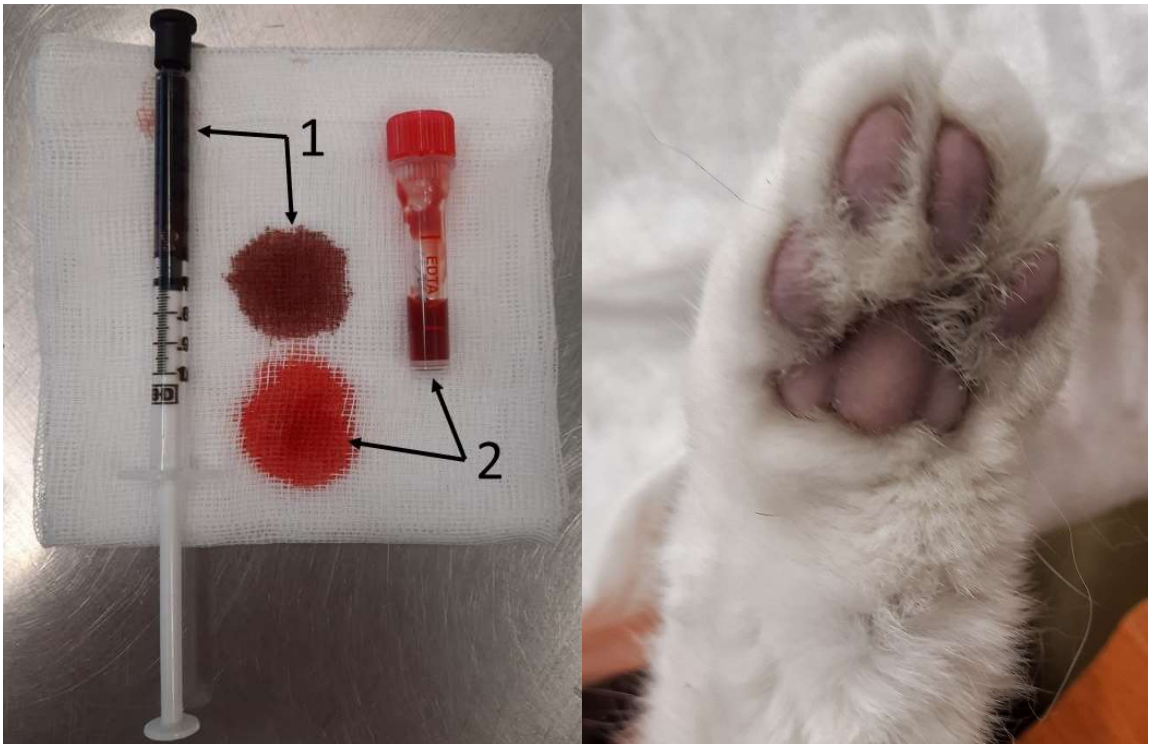

Figure S1 Left panel: MetHb spot-test for methemoglobin for the affected cat (1) and an unaffected cat (2). The blood of the affected cat keeps the brown discoloration even after being exposed to air. Right panel: cyanosis in the affected cat.

```
XP_044918404.1_CYB5R3_wt   MGAQLSTLGHVVLSPVWFLYNLFMKLFHRSTPAITLESPDIKYPLRLIDKEVINHDTRRF   60
CYB5R3_mut#1               MGAQLSTLGHVVLSPVWFLYNLFMKLFHRSTPAITLESPDIKYPLRLIDKEVINHDTRRF   60
CYB5R3_mut#2               MGAQLSTLGHVVLSPVWFLYNLFMKLFHRSTPAITLESPDIKYPLRLIDKEASTSTSRLE   60

XP_044918404.1_CYB5R3_wt   RFALPSPQHILGLPVG------------QHIYLSARIDGNLVIRPYTPVSSDDDKGFVDL  108
CYB5R3_mut#1               RFALPSPQHILGLPVGERSPDPARVEPGQHIYLSARIDGNLVIRPYTPVSSDDDKGFVDL  120
CYB5R3_mut#2               SMETWSSGPTLPSPAMTTRVLWTWSSRFTSKTPIPSFLLGARCPSTWKA-----------  109

XP_044918404.1_CYB5R3_wt   VIKVYFKDTHPKFPAGGKMSQYLESMKIGDTIEFRGPNGLLVYQGKGKFAIRPDKKSNPV  168
CYB5R3_mut#1               VIKVYFKDTHPKFPAGGKMSQYLESMKIGDTIEFRGPNGLLVYQGKGKFAIRPDKKSNPV  180
CYB5R3_mut#2               ------------------------------------------------------------  109

XP_044918404.1_CYB5R3_wt   IKTAKSVGMIAGGTGITPMLQVIRAIMKDPDDHTVCHLLFANQTEKDILLRPELEELRNE  228
CYB5R3_mut#1               IKTAKSVGMIAGGTGITPMLQVIRAIMKDPDDHTVCHLLFANQTEKDILLRPELEELRNE  240
CYB5R3_mut#2               ------------------------------------------------------------  109

XP_044918404.1_CYB5R3_wt   HSARFKLWYTVDKAPEAWDYSQGFVNEEMIRDHLPPPEEEPLILMCGPPPMIQYACLPNL  288
CYB5R3_mut#1               HSARFKLWYTVDKAPEAWDYSQGFVNEEMIRDHLPPPEEEPLILMCGPPPMIQYACLPNL  300
CYB5R3_mut#2               ------------------------------------------------------------  109

XP_044918404.1_CYB5R3_wt   DRVGHPKERCFTF                                                 301
CYB5R3_mut#1               DRVGHPKERCFTF                                                 313
CYB5R3_mut#2               -------------                                                 109
```

Figure S2 Amino acid alignment of the CYB5R3 wildtype protein (XP_044918404.1) and the two predicted mutant proteins, mut#1, XP_044918404.1:p.(G76_Q77insERSPDPARVEPG) and mut#2, XP_044918404.1:p.(V52Afs*58). The insertion of 12 additional amino acids in mut#1 is shown in blue. In mut#2, 58 amino acids that are altered by the frameshift are marked in red.
